# *In silico* analysis of glycosyltransferase 2 family genes in duckweed (*Spirodela polyrhiza*) and its role in salt stress tolerance

**DOI:** 10.1515/biol-2021-0063

**Published:** 2021-06-18

**Authors:** Mingliang Jiang, Peng Wang, Ligang Xu, Xiuxu Ye, Hongxiang Fan, Junxiang Cheng, Jinting Chen

**Affiliations:** Key Laboratory of Watershed Geographic Sciences, Nanjing Institute of Geography and Limnology, Chinese Academy of Sciences, Nanjing 210008, China; College of Resources and Environment, University of Chinese Academy of Sciences, Beijing 100049, China; Tropical Crops Genetic Resources Institute, Chinese Academy of Tropical Agricultural Sciences & Ministry of Agriculture Key Laboratory of Crop Gene Resources and Germplasm Enhancement in Southern China, No. 4 Xueyuan Road, Haikou 571100, Hainan, China

**Keywords:** *Spirodela polyrhiza*, dolichol phosphate mannose synthase 1, putative dolichyl-phosphate β-glucosyltransferase, gene structure, salt treatment

## Abstract

Plant glycosyltransferase 2 (GT2) family genes are involved in plant abiotic stress tolerance. However, the roles of GT2 genes in the abiotic resistance in freshwater plants are largely unknown. We identified seven GT2 genes in duckweed, remarkably more than those in the genomes of *Arabidopsis thaliana*, *Oryza sativa*, *Amborella trichopoda*, *Nymphaea tetragona*, *Persea americana*, *Zostera marina*, and *Ginkgo biloba*, suggesting a significant expansion of this family in the duckweed genome. Phylogeny resolved the GT2 family into two major clades. Six duckweed genes formed an independent subclade in Clade I, and the other was clustered in Clade II. Gene structure and protein domain analysis showed that the lengths of the seven duckweed GT2 genes were varied, and the majority of GT2 genes harbored two conserved domains, PF04722.12 and PF00535.25. The expression of all Clade I duckweed GT2 genes was elevated at 0 h after salt treatment, suggesting a common role of these genes in rapid response to salt stress. The gene Sp01g00794 was highly expressed at 12 and 24 h after salt treatment, indicating its association with salt stress resilience. Overall, these results are essential for studies on the molecular mechanisms in stress response and resistance in aquatic plants.

## 1 Introduction

Duckweeds are among the smallest, fastest-growing (the doubling time of the fastest-growing duckweeds under optimal growth conditions is <30 h) and morphologically simple flowering plants belonging to the early diverging phylum Magnoliophyta [1,2,3]. As one of the high-yield biomass species (up to 100 tons dry mass/hectare/year), duckweeds can be found on almost every continent except the Arctic and Antarctic regions, surviving in freshwater ponds and animal waste lagoons [4,5,6]. Duckweeds are highly adaptable to the environment. Many researchers have demonstrated that duckweed can grow well in many liquid media, including SH Hillman, Resh Hutner, WP, MS, N6, and B5 media [7]. They can adapt to a wide pH range, and most varieties can grow well within the pH range of 4.5–7.2 [7]. Many organic compounds (ethylene diamine tetraacetic acid (EDTA), citric acid, 2-morpholinoethanesulfonic acid (MES), and 3-(N-morpholino)propanesulfonic acid (MOPS)) and protein stabilizers (vinyl pyrrolidone (PVP)) have no significant effect on the growth of duckweed [8]. Duckweeds also exhibit antimicrobial resistance against waterborne fungi and bacteria [9,10].

The Lemnaceae family of duckweeds is taxonomically divided into 37 species across five genera [1,11]: *Spirodela*, *Lemna*, *Landoltia*, *Wolffia*, and *Wolffiella* [12,13]. *Spirodela polyrhiza* is a typical and easily surviving duckweed, with a water surface habitat composed of a tiny frond and a few adventitious roots (ARs) [6,12]. The fronds of the genus *Spirodela polyrhiza* are large, and their sticky roots help keep them upright in the water and spread by attaching to animals, rather than serving as an organ that primarily absorbs water and nutrients [6,14].

Salinity is one of the most severe global problems affecting agriculture. Saline stress causes vast economic and yield losses in agricultural production [15,16,17], as environmental salinity leads to growth inhibition, developmental changes, metabolic adaptation, and ion isolation or rejection [18]. In addition to soil salt stress, freshwater salt content has been rapidly increasing across broad regions of Asia, Europe, and North America, leading to significant effects in freshwater ecosystems [17,19,20]. The application of genetic engineering to improve varieties, thus improving the salt tolerance of plants, is a rapid and effective method to overcome salt stress in plants grown in freshwater and land [21]. In the past decade, research on the effect of salt on plant growth at the cellular level has mainly focused on the early signal transduction induced by salt [22]. Under salt stress, protein abundance is altered by gene expression, mRNA stability, and translation regulation. By targeting and phosphorylating various downstream targets such as SLAC1, KAT1, AtRbohF, and transcription factors required for the expression of numerous stress response genes, abscisic acid (ABA)-independent sucrose non-fermenting 1-related protein kinases (SnRKs) and ABA-dependent SnRKs play pivotal roles in transcriptional regulation and posttranscriptional regulation during abiotic stress responses [22,23,24]. Overexpression of NHX1 positively affects salt tolerance in some species, including tomatoes [22]. Among cereal crops, the expression of CIPK16 and AVP1 in barley improved salt tolerance under greenhouse and field conditions, respectively [25,26]. Furthermore, *Arabidopsis thaliana* ALG3 (Dol-P-Man: Man5GlcNAc2-PP-Dol alpha-1,3-mannosyltransferase) mutants were undistinguishable from the wild type when grown under normal or stress conditions, including salt stress and low and high temperatures [27]. The α-glucosylated N-glycan GlcM5ER, whose structure was modified with glucose residues using α-glucosyltransferases ALG6, ALG8, and ALG10, in yeast and *Arabidopsis thaliana*, was identified in ALG3 mutants. Furthermore, dolichol-phosphate glucose (Dol-P-Glc) is produced by dolichyl-phosphate beta-glucosyltransferase (ALG5), a member of the GT2 family [28]. Two resistance proteins, dolichol phosphate mannose synthase 1 (DPMS1) and ALG5, belong to the GT2 family and might be worthy of further attention concerning plant salt tolerance. DPMS1 significantly matches (BLASTP, *E* value = 9 × 10^−47^) the peptide sequence encoded by At1g20575; it influences development, stress response, and ammonium hypersensitivity, mediates isoprenyl-linked glycan biogenesis, and is localized in the ER [29]. It encodes the catalytic core of the dolichol phosphate mannose synthase (DPMS) complex and is not active on its own but requires the presence of DPMS2 and DPMS3 for full activity [29]. DPMS1 is located in the endoplasmic reticulum and endoplasmic reticulum membrane and is expressed during most growth periods and in most organs of plants, contributing to dolichyl-phosphate beta-d-mannosyl transferase activity [30,31]. ALG5 is encoded by the AT2G39630 gene in *Arabidopsis thaliana*, localizes in the endoplasmic reticulum, endoplasmic reticulum membrane, and extracellular regions, and is expressed during the growth and developmental stages in most plant structures [30,31,32].

*Spirodela polyrhiza* has a genome size of 158 Mb, one of the smallest duckweeds, similar to the plant model genome of *Arabidopsis thaliana* [3,33]. The initial *Spirodela polyrhiza* reference generated by the Department of Energy Joint Genome Initiative (DOE-JGI) was based on clone 7498 (Sp7498) collected from Durham, North Carolina, USA (35 N 75 W) [34]. An initial reference genome draft with approximately 20× coverage for the *Spirodela polyrhiza* strain 7498 (Sp7498) was published, revealing that it has the fewest predicted genes of any known plant genomes, at 19,623 [3]. The Sp7498 genome assembly only covered 90% of the expected genome size, and 10.7% of the assembly in gaps were filled with Ns [13]. Instead of being resolved into the expected 20 chromosomes, the genes were anchored onto 32 pseudomolecules [3]. To overcome these limitations and obtain genome-wide information on the intraspecific variations between different *Spirodela polyrhiza* populations, Michael et al. combined high-depth short-read sequencing and high-throughput genome mapping technologies for comprehensive studies on the genome of *S. polyrhiza* strain 9509 (Sp9509) [35]. They found that *Spirodela polyrhiza* has the lowest levels of genome-wide DNA methylation among any of the flowering plants examined. The Sp9509 assembly was later updated using Oxford Nanopore single-molecule long-read sequencing, and the chromosomal structure was validated using orthogonal techniques [12]. Recently, the Sp7498 genome was updated using Pacific Biosciences (PacBio) Sequel reads, which can reach a 44-fold increase in contiguity with an N50 (a median of contig lengths) of 831 kb, which filled 95.4% of the gaps in the previous version; this version has been used for studying the evolution and environmental adaptation of *Spirodela polyrhiza* [6]. In their latest study, Harkess et al. generated a highly contiguous, chromosome-scale assembly of *Spirodela polyrhiza* line Sp7498 using Oxford Nanopore plus Hi-C scaffolding (Sp7498_HiC) and found that both the Sp7498_HiC and Sp9509 genome assemblies had large chromosomal misorientations from a recent PacBio assembly of Sp7498, highlighting the necessity of orthogonal long-range scaffolding techniques such as Hi-C and BioNano optical mapping [13]. This is the most accurate and contiguous *Spirodela polyrhiza* genome available to date.

Investigating the gene repertoire in freshwater plants can provide insights on salt tolerance in freshwater, which can be transferred to other crops [36]. To this end, focusing on genetically tractable duckweed with high-quality genome information may help us exploit the natural salt tolerance of halophytes for crop improvement. Considering its strong environmental adaptability, stress resistance, and dominant plant expression system, purple duckweed was selected as the research object in this study. Despite the significant role of this species, few molecular investigations have been undertaken for duckweed, and even though several versions of its genome assembly have been released, utilization of this genome information has only just begun. In particular, few systematic identification characteristics of a given gene family have been determined in the genome of this species. In this study, GT2 family members were identified genome-wide using the newly released duckweed genome. The biological expression of duckweed DPMS1 and ALG5 genes was analyzed to understand the characteristics of their salt reactions and improve the biological yield of duckweed by optimizing different salt reactions. To our knowledge, this is the first systematic identification and analysis of the GT2 family in duckweed and will have implications for studies on the molecular mechanisms of stress response and resistance in aquatic plants.

## 2 Materials and methods

### 2.1 Data sources and sequence collection

Datasets of putative proteins and coding sequences were obtained from the sources as described by Wang et al. [37]: *Arabidopsis thaliana*: TAIR [38]; *Oryza sativa*: http://rice.plantbiology.msu.edu [39], *Spirodela polyrhiza*, *Amborella trichopoda*, *Nymphaea tetragona*, *Persea americana* (*avocado*), *Zostera marina*, and *Ginkgo biloba*: Phytozome v13.0 [6,40,41]. The data are preprocessed through our internal Perl scripts to remove short sequences, simplify the entry lines of sequence items, and remove symbols such as asterisks (*) that can interfere with subsequent data processing [42].

*Arabidopsis thaliana* sequences containing GT2 domains were first retrieved from NCBI and then the TAIR dataset was BLAST searched to generate the nonredundant DPMS1 sequence dataset which was used as the query sequence of BLAST search for other plant genomes in this study. An independent peptide sequence similarity search was conducted using BLASTP, an NCBI BLAST + executable suite [43]. In addition, the “trusted cutoff” was used as the threshold to detect the DPMS1 and ALG5 domains (Pfam Clan ID: PF00535, PF01715) to conduct an HMM search for the protein dataset. The results of the above two searches were merged into a primitive sequence file that was used to search a family library of Pfam-A using pfam_scan.pl, a Perl program downloaded from the Pfam ftp site (http://sanger.ac.uk/), with minor modifications, and e_seq = 1e–2 and e_dom = 1e–4. The output items with PF00535 and PF01715 as CLAN names and less than 1 significance were retrieved. Locus IDs are retrieved if multiple locus IDs represent multiple gene models of the same loci; only the one with the longest transcript is retained. The coding region and peptide sequences of each locus ID were retrieved. DNA sequences were visually examined to avoid regional large fragment deletions in the DPMS1 or ALG5 domains.

### 2.2 Sequence and phylogenetic analyses

The peptide sequences were aligned using Probcons 1.12, and DNA sequences were performed using MUSCLE 3.8.31. Alignment results were visualized using the BioEdit V7.2. The FASTA format of the alignment file was converted to NEXUS or PHYLIP formats through our internal Perl scripts, if needed.

Maximum likelihood phylogenetic trees were reconstructed with PhyML software using a bootstrap method with 1,000 replicates [44]. The standard deviation of the split frequencies was checked after each run of 100 million generations to ensure that they were below 0.05 [37,42]. Unless otherwise noted, the parameters for phylogenetic inference were set to the default values. Phylogenetic trees were visualized using FigTree v1.4.4 (http://tree.bio.ed.ac.uk/software/figtree/).

A dot in the Seq-Tools suite was used for dot plot analysis [45]. The genetic distance between populations was calculated using the MEGA 5.08 [46]. SPSS V25.0 was used for the statistical analysis.

### 2.3 Nomenclature of gene models

Based on the phylogenetic analysis of all GT2 gene members in this study, the gene models were named DPMS1, ALG5, and GDP-mannose. If there was more than one member of the same species in each group, the names were suffixed with Roman numerals. The locus ID was then fixed for each gene model. In addition, unless otherwise stated, each gene model was prefixed with the initial capital letter of genus name and the first or first two letters of the species name (*Arabidopsis thaliana*: AT, *Oryza sativa*: Os, *Spirodela polyrhiza*: Sp, *Amborella trichopoda*: scaffold, *Nymphaea tetragona*: Nco, *Persea americana* (*avocado*): Pam, *Zostera marina*: KMZ, and *Ginkgo biloba*: Gb). For example, AT1G20575.1 represents *the Arabidopsis thaliana* gene, whose locus ID is AT1G20575.1.

### 2.4 Public microarray-based expression analysis of *Spirodela polyrhiza* genes

In order to study the gene expression patterns in different duckweed tissues and treatments, we used “*Spirodela polyrhiza*” as the searching ID to download publicly available microarray data standardized for multi-array averaging (RMA) from NCBI. A heatmap was drawn using the pheatmap, an R package.

## 3 Results

### 3.1 Identification and phylogenetic analysis of glycosyltransferase 2 (GT2) family genes in plants

**Figure 1:**
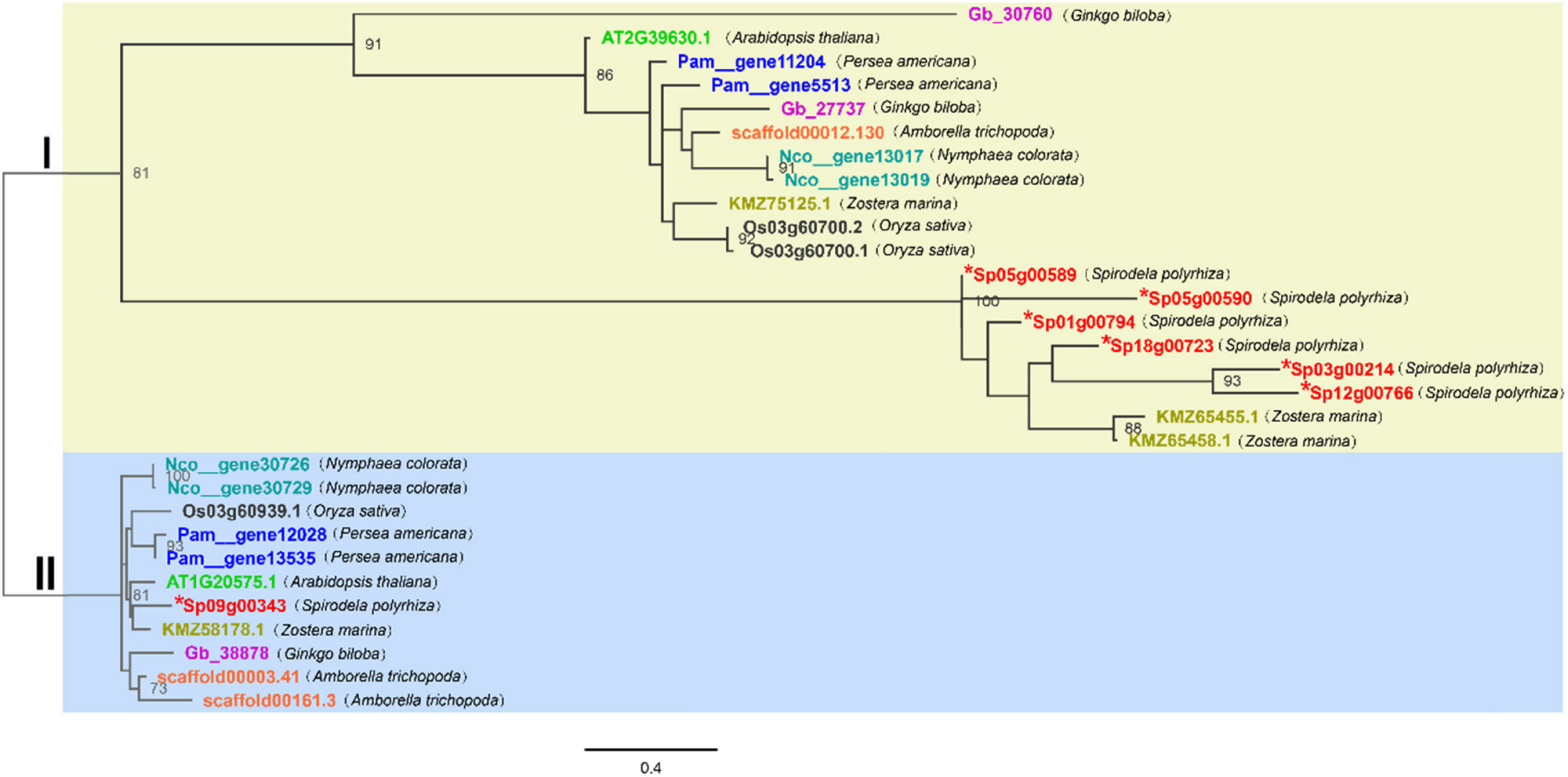
Phylogenetic relationships of GT2 genes in different plants. Maximum likelihood phylogeny was used. The tree was constructed using PhyML with 1,000 bootstrap replicates. The numbers beside the nodes indicate the bootstrap values for every 100 replicates. The scale shows the genetic variation for the length of the scale: The value of 0.4 represents a 40% difference between the two sequences. In this study, two *Arabidopsis thaliana*, three *Ginkgo biloba*, three *Oryza sativa*, seven *Spirodela polyrhiza*, three *Amborella trichopoda*, four *Persea americana*, four *Nymphaea tetragona*, four *Zostera marina*, and a total of 30 full-length peptide sequences were identified and analyzed. The genes of different species are shown in different colors. The scales measure the evolutionary distance in substitutions per amino acid. Sequences were aligned using MUSCLE. The tip labels show gene names: gene name prefix letters represent different species: Gb *ginkgo biloba*, AT *Arabidopsis thaliana*, Pam *Persea americana*, scaffold *Amborella trichopoda*, Nco *Nymphaea tetragona*, KMZ *Zostera marina*, Os *Oryza sativa*, and Sp *Spirodela polyrhiza.* * indicates *Spirodela polyrhiza* GT2.

There are two known members of *the Arabidopsis thaliana* GT2 gene family: DPMS1 (locus ID: AT1G20575.1) and putative ALG5 (locus ID: AT2G39630.1) [29,31,32]. To investigate the distribution of GT2 gene families across plant clades, we analyzed the GT2 gene families of representative plants whose genomes are publicly available, including one gymnosperm, four dicotyledon species, and three monocotyledon species. First, we queried the peptide sequences of *Arabidopsis thaliana* GT2, searched the sequencing datasets of eight genomes using BLASTP and HMM methods, and then screened the data through a series of steps, as described in Section 2. In total, we identified 30 GT2 gene models from the above genomes, including *Arabidopsis thaliana*. Among the 30 gene sequences, two genes of *Arabidopsis thaliana*, three genes of *Oryza sativa*, and four genes of *Zostera marina* are known to have functional characteristics. To analyze the phylogenetic relationship of GT2 group members in detail, maximum likelihood phylogenetic analysis was performed (Figure 1). Among the plant gene models, in the genome of *Arabidopsis thaliana*, only two GT2 gene models have been identified, while in the genome of *Spirodela polyrhiza*, at least seven GT2 genes are known. As shown in Figure 1, the plant GT2 genes were split into two distinct clades, namely ALG5 (Clade I) and DPMS1(Clade II). Clade I is further divided into two branches, ALG5 and a new gene protein, glucomannan 4-beta-mannosyltransferase (GDP-Man) [47]. Genes in Clade I were more populated than those in Clade II, where Clade Ⅰ had 19 gene models and Clade II had 11. Nevertheless, all the plants we investigated harbored these two genes in the genomes.

Six genes were present in Clade I, which encode ALG5 and dolichyl-phosphate beta-d-mannosyltransferase/glucomannan 4-beta-mannosyltransferase (GDP-mannose, GDP-Man). ALG5, which is localized on the rough endoplasmic reticulum (rER) membrane, catalyzes the transfer of glucose from UDP-glucose (UDP-Glc) to dolichyl-phosphate (Dol-P) to synthesize dolichol-phosphate glucose (Dol-P-Glc, DPG, Glc-P-Dol, or GPD). Therefore, this enzyme is also known as the Dol-P-Glc synthase [47]. Among these six genes, three GDP-Man genes from *Zostera marina* [41], one ALG5 gene from *Arabidopsis thaliana*, and two ALG5 genes from *Oryza sativa* [48], which had been previously identified and characterized, were included in this group.

Three genes previously identified were present in Clade II, one in *Arabidopsis thaliana* [47], one in *Oryza sativa* [49] and one in *Zostera marina* [41]. They encode DPMS1. The dolichyl-phosphate mannosyltransferase polypeptide catalyzes the transfer of mannose from GDP-mannose (GDP-Man) to dolichyl-phosphate (Dol-P) to produce dolichol-phosphate mannose (Dol-P-Man or DPM). Hence, it is also called DPM synthase (DPMS) [28,29].

In Clade I, six duckweed full-length peptide sequences were present on the same branch [41,48], suggesting that the ALG5 gene of *Spirodela polyrhiza* had various functions, but the evolutionary relationship was similar. The phylogeny also showed that a single-copy gene encoded the GDP-Man of ALG5 in *Spirodela polyrhiza*. In Clade I, *Spirodela polyrhiza* genes and two genes of *Zostera marina* (KMZ65455.1 and KMZ65458.1) were on separate branches, suggesting that they have similar functions and evolutionary history.

Compared with *Spirodela polyrhiza*, both *Arabidopsis thaliana* and *Amborella trichopoda* have only one gene in Clade I. Except for *Zostera marina*, which has three copies, ALG5 genes likely experienced two duplication events in the early stage of the evolution of all other plants, suggesting that they have more conservative functions. The genes of *Oryza sativa* and *Nymphaea tetragona* were evolutionarily close to each other in this clade, indicating that they have similar functions. On the contrary, the gene function of *Gingko biloba* might vary considerably.

In Clade II, only one gene is represented in each of the species investigated, including duckweed, which indicates that the roles of DPMS1 genes are conserved in plants. Furthermore, there is only one DPMS1 gene in *Ginkgo biloba*, *Zostera marina*, and *Oryza sativa*, whereas there are two or three genes in the ALG5 group. In Clade I, relative to other plants, the genes of *Ginkgo biloba* (Gb_30760 with Gb_27737) and the genes of *Zostera marina* (KMZ75125.1, KMZ65455.1, and KMZ65458.1) are far apart, whereas the genes of the same plants are very close together in Clade II. That also proved that the DPMS1 genes in plants are conserved compared to the ALG5 genes.

### 3.2 Gene structure and protein domains of *Spirodela polyrhiza* GT2 genes

**Figure 2:**
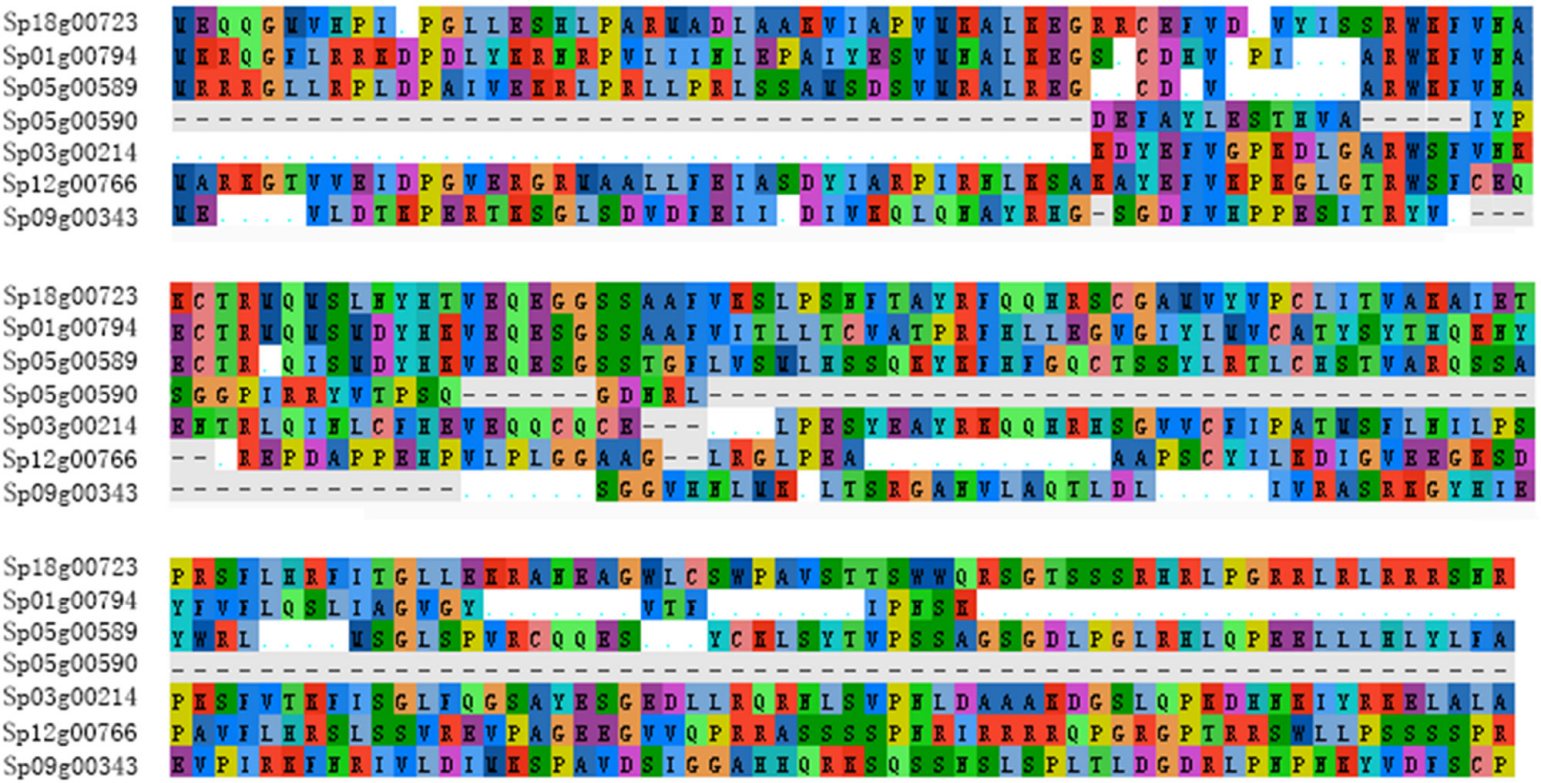
Multiple sequence alignment of the seven GT2 genes in *Spirodela polyrhiza*. Alignment of the deduced amino acid sequences of Sp18g00723, Sp01g00794, Sp03g00214, Sp12g00766, Sp05g00589, Sp05g00590, and Sp09g00343 by using Mega software. Different letters and colors denote different amino acids.

**Figure 3:**
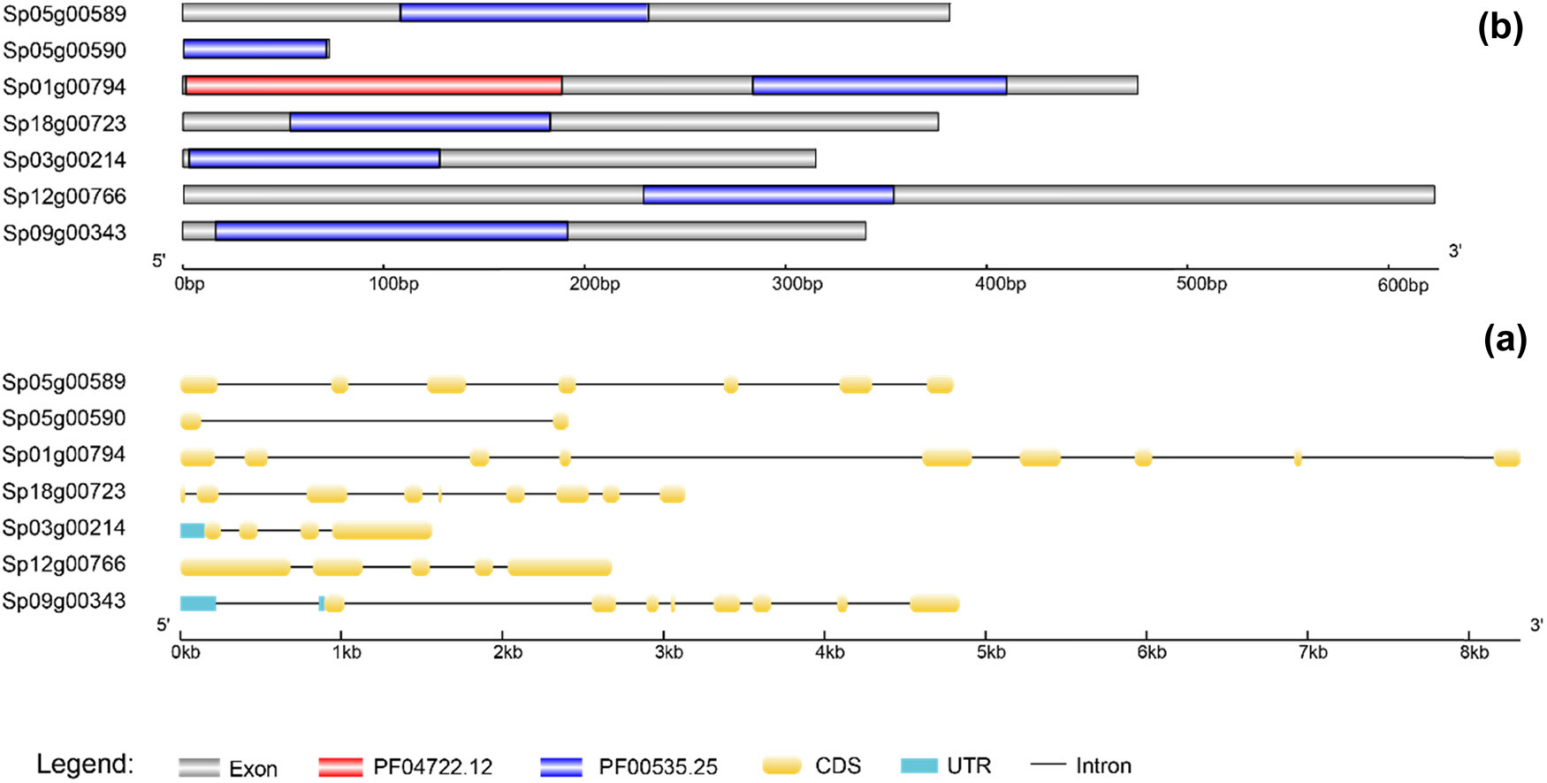
Structure and conserved domain map of the *Spirodela polyrhiza* GT2 gene. (a) The structure of *Spirodela polyrhiza* GT2 genes. Light blue rectangles, yellow rounded rectangles, and black lines represent the UTR, CDS, and introns, respectively. The scale bar on the bottom estimates the length of the UTR, CDS, and introns. UTR indicates an untranslated region. (b) The structure of the *Spirodela polyrhiza* GT2 protein. Blue and purple rectangles represent the PF00535.25 and PF04722.12 domains. The scale bar on the bottom estimates the length of PF00535.25 and PF04722.12.

Using Mega software, multiple sequence alignment of the seven GT2 genes in Spirodela polyrhiza was done (Figure 2). We also mapped the structure and conserved domains of these genes (Figure 3). Furthermore, remarkable differences in the gene structures were observed among the plant GT2 genes. The information of the GT2 genes in *Spirodela polyrhiza* is listed in Table 1. The nucleic acid (genomic DNA) length of these genes ranged from 1,564 to 8,320 bp, the CDS length ranged from 222 to 1,875 bp, and their average values were 3,964 and 1,114 bp, respectively. The number of exons was 2–9 and the number of introns was 1–8. Sp05g00590 had the shortest CDS length and the lowest CDS number and intron number (2 and 1, respectively). The Sp09g00343 gene had the second longest gene length, but its CDS length was only 74 bp longer than that of Sp03g00214 which has the shortest gene length. Comparing the *Spirodela polyrhiza* GT2 genes, the ratio of CDS length to gene length was the largest for Sp12g00766, with a value of 69.8%, followed by Sp03g00214, whose value was 60.7%, and the minimum was observed for Sp05g00590 (9.3%). For Sp01g00794, which had the longest gene length, the ratio of CDS to gene length was 17.2%.

**Table 1:** Information of the GT2 genes in *Spirodela polyrhiza*

Gene ID	Chromosome (Chr)	Start–end in Chr.	Strand	CDS length (bp)	Isoelectric point (pI)	Molecular weight (MW kDa)	Exons
Sp18g00723	chr18	162,230—165,367	−	1,134	10.52	43.4	9
Sp01g00794	chr1	5,490,338–5,498,658	+	1,434	6.38	54.2	9
Sp03g00214	chr3	7,737,441–7,739,005	+	951	8.27	36.7	4
Sp12g00766	chr12	4,933,193–4,935,871	−	1,875	10.19	68.3	5
Sp05g00589	chr5	3,888,592–3,893,391	+	1,152	9.36	43.4	7
Sp05g00590	chr5	3,884,599–3,887,009	+	222	4.89	8.5	2
Sp09g00343	chr9	1,442,094–1,446,931	−	1,029	8.87	38	9

Similar to other species, duckweed GT2 gene family members only have two domains, PF04722.12 and PF00535.25 (Figure 3b). Only Sp01g00794 has a PF04722.12 domain, and the other members have only the PF00535.25 domain. Domain PF00535.25, a member of the superfamily cl11394, is used to transfer sugar from UDP-glucose, UDP-N-acetyl-galactosamine, GDP-mannose, or CDP-abequose, to a range of substrates including cellulose, dolichol phosphate, and teichoic acids [50]. The conserved protein domain PF04722.12 refers to Ssu72-like protein. The highly conserved and essential protein Ssu72 has intrinsic phosphatase activity and plays an essential role in the transcription cycle [51,52]. Sp01g00794 has both the conserved protein domain family PF04722.12 and PF00535.25, which are located at 3–191 aa and 285–412 aa, respectively. The locations of PF00535.25 in Sp18g00723, Sp03g00214, Sp12g00766, Sp05g00589, Sp05g00590, and Sp09g00343 are 55–185 aa, 5–129 aa, 230–356 aa, 110–233 aa, 1–73 aa, and 18–193 aa, respectively.

### 3.3 Expression of GT2 genes in the leaves and roots of duckweed

**Figure 4:**
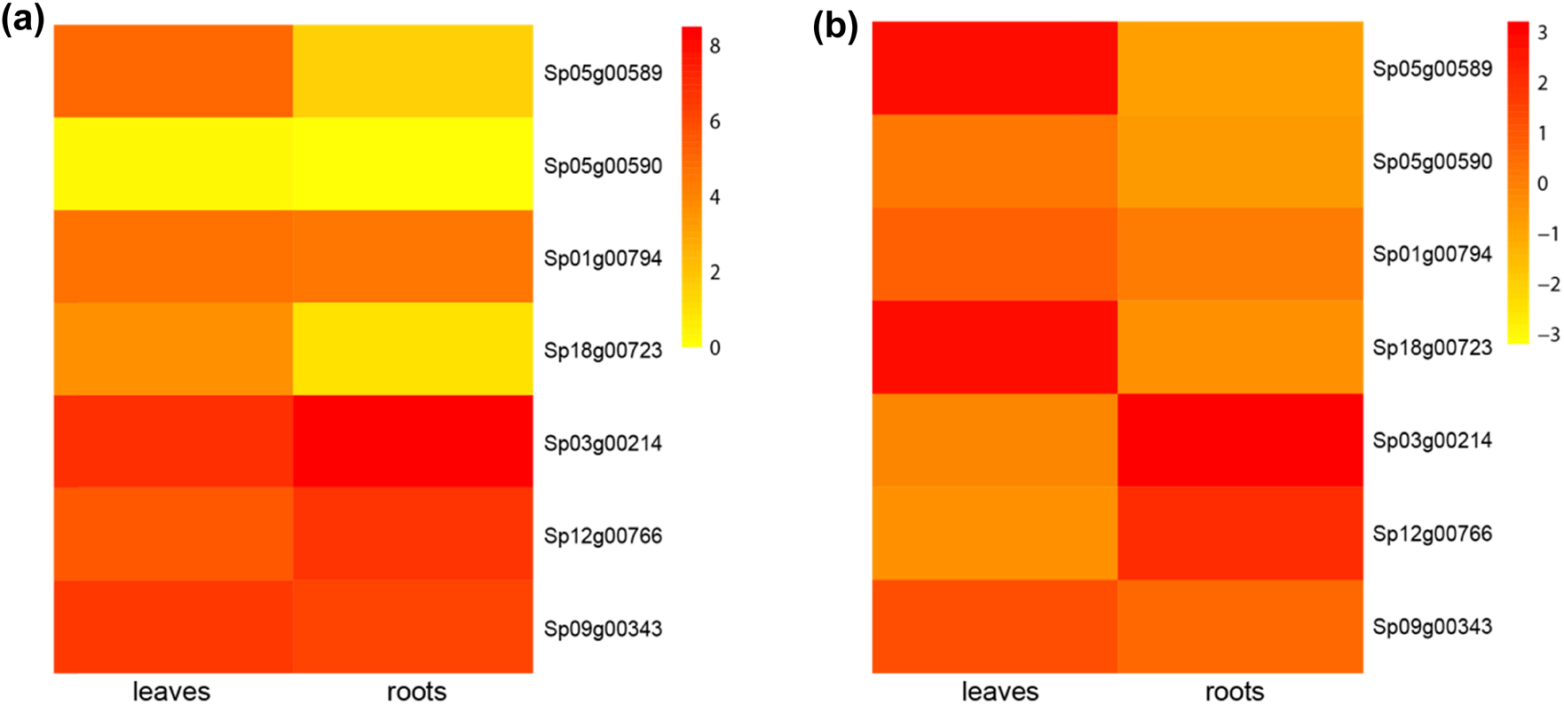
Expression of GT2 genes in the leaves and roots of duckweed. (a) Heatmap of the duckweed GT2 gene expression. (b) Heatmap of the normalized expression of duckweed GT2 genes, showing the relative expression between the leaves and roots for each gene.

As an aquatic plant, duckweed floats on the water’s surface, with leaves and roots constituting the major vegetative organs. We examined the expression of GT2 genes using public transcriptomic data. Sp05g00590 was not expressed in either the leaves or roots (Figure 4a). Considering that the peptide sequence of this gene is unusually short (Figure 3), the gene Sp05g00590 is probably pseudogenized. Sp03g00214 is highly expressed in both leaves and roots, suggesting a constitutive role for this gene. Sp05g00589 and Sp18g00723 were highly expressed in leaves, whereas Sp12g00766 was highly expressed in the roots, indicating that the functions of these genes were differentiated. The Clade II gene, Sp09g00343, is highly expressed in both the leaves and roots, indicating the constitutive role of this Clade II gene in duckweed.

### 3.4 Expression of duckweed GT2 genes in response to salt stress

**Figure 5:**
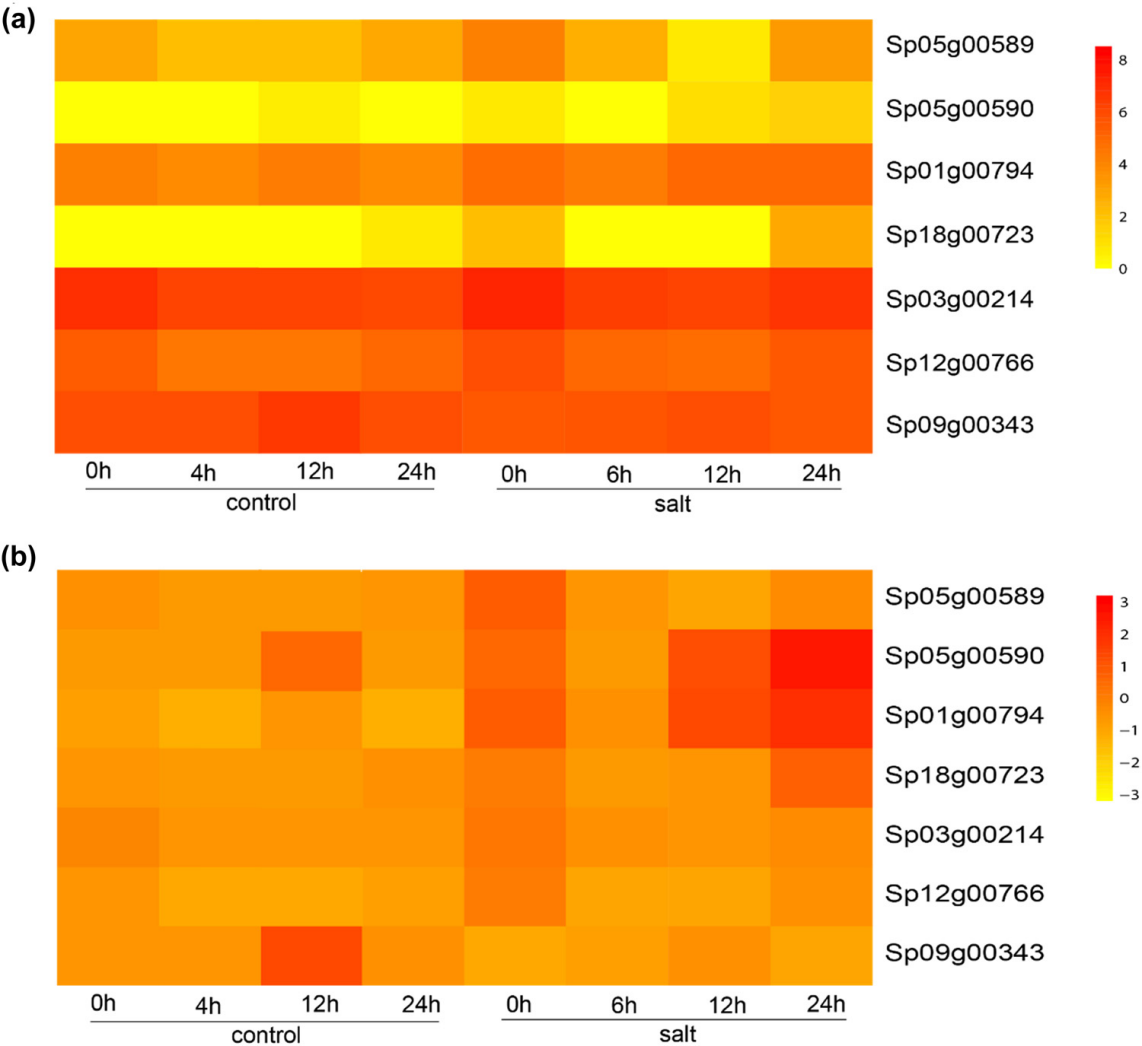
Expression of GT2 genes in response to salt treatment in duckweed. (a) Heatmap of the duckweed GT2 gene expression. (b) Heatmap of the normalized expression of duckweed GT2 genes, showing relative expression between the leaves and roots for each gene.

Expression of all clade I GT2 genes was elevated at 0 h after salt treatment, suggesting a rapid response of all Clade I genes to salt stress (Figure 5). However, their expression was decreased at 6 h after treatment; at 12 and 24 h, the expression of Sp01g00794 was increased, indicating that this gene is responsible for salt resilience. Sp18g00723 was also expressed at low levels, indicating that this gene is not associated with salt response. The short gene Sp05g00590 was expressed at extremely low levels, corroborating its pseudogenization. No obvious change was observed for the Clade II gene Sp09g00343 with salt treatment, suggesting that the Clade II GT2 gene is not associated with salt response (Figure 5).

## 4 Discussion

In this study, we obtained a total of 30 GT2 genes from eight plants. Nine of the 30 genes had been identified and characterized. These genes encode dolichyl-phosphate beta-d-mannosyltransferase (KMZ75125.1 and KMZ58178.1), putative ALG5 (AT2G39630.1, LOC_Os03g60700.1, and LOC_Os03g60700.2), glucomannan 4-beta-mannosyltransferase (KMZ65458.1 and KMZ65455.1), and DPMS1 (LOC_Os03g60939 and AT1G20575), respectively.

Mannose residues transported by Dol-P play a key role in the biosynthesis of *N*-glycoproteins, *O*- and *C*-mannosylated proteins, and GPI-anchored proteins. In addition, removal of the terminal Man residue donated by Dol-P-Man is the last enzymatic step before glycoproteins can exit the ER. If folding is not completed at this step, the attached glycan dictates degradation of the polypeptide chain [53,54,55]. The dolichyl-phosphate mannosyltransferase polypeptide catalyzes the transfer of mannose from GDP-mannose (GDP-Man) to dolichyl-phosphate (Dol-P) to produce dolichol-phosphate mannose (Dol-P-Man or DPM). Although this enzyme is widely conserved among eukaryotes, its existing form is mainly divided into two classes: in budding yeast, fungi, and protozoa, dolichyl-phosphate mannosyltransferase exists as one polypeptide (Dpm1p), whereas it consists of three polypeptides (DPM1-3) in fission yeast, worms, and vertebrates [56,57]. Jadid et al. also provided evidence that *Arabidopsis thaliana* DPMS1, DPMS2, and DPMS3 are functional orthologs of mammalian genes involved in Dol-P-Man synthesis [29]. This enzyme belongs to GT2 family based on Carbohydrate-Active enZymes (CAZy) classification, which includes ALG5 and hyaluronan synthase 1–3 (HAS1-3).

Previous studies on plant DMPS1 mainly used *Arabidopsis thaliana* as a model [29,58,59], and the results also showed that DPMS1 genes were localized in the ER and mediated isoprenyl-linked glycan biogenesis, which is widely involved in the regulation of plant growth and development as well as anti-stress regulation. It has been shown in yeast that changes in protein glycosylation pathways alone lead to the aggregation of misfolded proteins in the ER lumen, leading to ER stress. During ER stress, the most significant change occurred in the transcriptional induction of genes that control the unfolded protein response pathway [60]. Mutations in the DPMS1 gene encoding the catalytic module led to a distinct phenotype characterized by widespread greening, reduced root growth, and seed coat wrinkling under normal culture conditions. Loss-of-function mutations and RNA interference-mediated reduction of DPMS1 expression in *Arabidopsis thaliana* also caused a wrinkled seed coat phenotype and remarkably enhanced hypersensitivity to ammonium, which was manifested by extensive chlorosis and a substantial reduction in root growth.

Unlike *Arabidopsis thaliana*, *Spirodela polyrhiza* is an aquatic plant with fewer predicted genes compared to most known plant genomes. Duckweed has good resistance to environmental stress. The crude extract of duckweed has antibacterial properties against waterborne fungi and bacteria [6,10], and chloroplasts in the duckweed species, *Lemna trisulca*, are mobile in response to heavy metals [61], suggesting that chloroplast dynamics in duckweeds may favor the ability of rapid spatial reorganization in response to environmental stress [13]. Although the functions of the seven genes of *Spirodela pol*y*rhiza* studied in this article have not been identified, we can speculate their functions according to the research results. Phylogenetic analysis of GT2 family genes showed that *Spirodela polyrhiza* had both DPMS1 and ALG5 genes in the GT2 family. Furthermore, the evolution of ALG5 was closely related to that of the marine plant *Zostera marina* genes (KMZ65458.1 and KMZ65455.1), which encodes glucomannan 4-beta-mannosyltransferase (GDP-Man). This suggests that the *Spirodela polyrhiza* genes in Clade I may belong to ALG5 and mainly encode GDP-Man, an underlying protein that produces DPM. There are six copies of *Spirodela polyrhiza* genes on one branch of the evolutionary tree, indicating that this *Spirodela polyrhiza* gene has various functions. In this study, seven GT2 family members selected from the genome of *Spirodela polyrhiza* showed certain differences in gene structure and protein conserved domains. The exon and intron arrangement patterns were different. Based on the gene structure and expression level, we determined that Sp05g00590 may be a pseudogene. In addition, the expression characteristics of *Spirodela polyrhiza* to salt stress indicated its good adaptability to salt stress. The *Spirodela pol*y*rhiza* gene Sp09g00343 was grouped in Clade II, a DPMS1 gene group, indicating that the *Spirodela polyrhiza* can cope with environmental stress.

We observed significant expression induction of Clade I among the GT2 genes in duckweed by salt stress, with a rapid response of expression elevation after the treatment, which indicates that the Clade I genes of the family are responsible for the rapid response to salt stress in duckweed. An *Arabidopsis thaliana* gene (At2g39630) is phylogenetically clustered with clade I genes, which is putatively a dolichyl-phosphate glucosyltransferase. However, its biochemical functions and role in the salt stress response of *Arabidopsis thaliana* have not been investigated yet. Our results indicate that Clade I genes of the GT2 family are involved in the early response to salt stress. Moreover, their functions have been reinforced in duckweed as suggested by the expansion of these genes in the duckweed genome. Some of the genes were elevated at 12 and 24 h after stress, indicating that some Clade I genes might be involved in the late response to salt stress in duckweed. Thus, it is necessary to further investigate their exact roles in salt stress response by duckweed using molecular and biochemical tools, which are largely limited by the lack of molecular platforms such as tissue culture and transformation for this species. To our surprise, information on the Clade I genes of *Arabidopsis thaliana* is limited for its biochemical and biological functions, which should be addressed in future studies.

For expression analysis, we used the publicly available RNASeq data. We noticed a discrepancy in sampling time. In the controls, plants were sampled at 4 h and samples were taken at 6 h after treatment. That may introduce bias or even errors in data interpretation. However, the sampling times are consistent for the other three time points, and the overall trend supports our argument regarding their roles in salt stress response. Future studies should use qRT-PCR and *in vitro* or *in vivo* transgenic approaches to further confirm the roles of these genes in stress response.
